# Identification of *Vibrio metschnikovii* and *Vibrio injensis* Isolated from Leachate Ponds: Characterization of Their Antibiotic Resistance and Virulence-Associated Genes

**DOI:** 10.3390/antibiotics12111571

**Published:** 2023-10-28

**Authors:** Aura Falco, Miguel Ángel Villaquirán-Muriel, José David Gallo Pérez, Alejandra Mondragón-Quiguanas, Carlos Aranaga, Adriana Correa

**Affiliations:** 1 Microbiology, Industry and Environment Research Group (GIMIA), Department of Basic Sciences, Santiago de Cali University, Cali 760035, Colombiaadriana.correa00@usc.edu.co (A.C.); 2 Chemistry and Biotechnology Research Group (QUIBIO), Department of Basic Sciences, Santiago de Cali University, Cali 760035, Colombia; carlos.aranaga00@usc.edu.co

**Keywords:** *Vibrio metschnikovii*, *Vibrio injensis*, antibiotic, resistance, virulence, beta-lactams

## Abstract

This study aimed to evaluate the antibiotic resistance of 22 environmental *Vibrio metschnikovii* isolates and 1 *Vibrio injensis* isolate from landfill leachates in southwestern Colombia. Isolates were identified by Matrix-Assisted Laser Desorption/Ionization–Time-Of-Flight (MALDI-TOF), and 16S ribosomal RNA gene sequencing. Analysis of the susceptibility to six antibacterial agents by the Kirby–Bauer method showed susceptibility of all the isolates to ciprofloxacin and imipenem. We recorded resistance to beta-lactams and aminoglycosides, but no multidrug resistance was observed. The genome of one of the isolates was sequenced to determine the pathogenic potential of *V. injensis*. Genes associated with virulence were identified, including for flagellar synthesis, biofilm formation, and hemolysins, among others. These results demonstrate that landfill leachates are potential reservoirs of antibiotic-resistant and pathogenic bacteria and highlight the importance of monitoring *Vibrio* species in different aquatic environments.

## 1. Introduction

Leachates occur when rainwater and humidity percolate through garbage stored in sanitary landfills, thereby extracting contaminants and generating a liquid that may potentially damage the environment [1] and human and animal health [2]. Leachates are composed of organic matter, inorganic components, and xenobiotic compounds such as antibiotics [1,2,3], making them one of the most concerning sources of global contamination as they pose a potential risk to the health of the ecosystems involved in their discharge [4,5]. Song et al. [6] highlighted the need for research on both landfills and leachates because these stimulate the selection and proliferation of antimicrobial-resistant bacteria. Furthermore, they suggested that antibiotic-resistance genes (ARG) transferred between bacterial populations pose a risk to public health, and they point out that the data obtained from this type of research are essential for developing strategies, action plans, and risk management in possible adverse scenarios for human, animal, and environmental health (One Health context) [6,7].

The genus *Vibrio* belongs to the family *Vibrionaceae*, which includes a group of bacteria that are natural inhabitants of aquatic environments, including freshwater, estuarine, and marine environments [8]. Several species in this genus can be pathogenic for humans, and *Vibrio cholerae* is the most widely studied. The serogroups O1 and O139 cause outbreaks of cholera. This disease is characterized by clinical symptoms including acute diarrhea, which can affect children and adults and—in the case of severe disease—can be fatal [9]. Infections caused by *Vibrio* other than *Vibrio cholerae* are called “vibrioses” [8] and can range from mild cases of gastroenteritis to life-threatening conditions such as sepsis and invasive skin and soft tissue infections [10]. *Vibrio parahaemolyticus* is the most prevalent species that causes vibriosis associated with the consumption of fish and shellfish [11]. Furthermore, *V. vulnificus* is an opportunistic pathogen that causes wound infections that can rapidly lead to septicemia, which is the reason why it is associated with high mortality [12]. Other species—such as *Vibrio fluvialis*, *Vibrio mimicus*, and *Vibrio metschnikovii*—have lower incidence rates. However, the number of cases associated with these species has increased over the past few years [13,14,15,16,17], which indicate that they are a potential source of concern.

Even though the *Vibrio* species are usually susceptible to antibiotics in clinical settings, several studies have reported their resistance [8,18,19,20,21,22]. Mechanisms of antimicrobial resistance identified in *V. cholerae* include efflux pumps, modification of target proteins, and genes involved in the production of enzymes that hydrolyze or modify antibiotics [23]. These mechanisms have also been reported for other *Vibrio* species [20]. A recent research showed that wild birds can be important disseminators of species of the genus *Vibrio* that carry resistance genes [20]. The objective of this study was to identify and determine the antibiotic susceptibility profile of 22 *Vibrio* isolates collected from leachates from a leachate treatment plant (LTP) located in Cali, Colombia, due to the importance of leachates as a source of antimicrobial-resistant environmental pathogens. Additionally, we characterized the genes associated with virulence and resistance to antibiotics of an isolate identified as *Vibrio injensis*, which involved genome sequencing to determine its pathogenic potential.

## 2. Results

### 2.1. Collection of Leachate Samples

We obtained 23 sucrose-fermenting isolates, i.e., yellow colonies on TCBS agar. Of these, 5 (22%) and 18 (78%) isolates with this phenotype were obtained from Lagoon 3 and 7, respectively (Table 1).

### 2.2. Identification of the Isolates of Vibrio *spp.*

All the isolates obtained (*n* = 23) were characterized as Gram-negative bacilli with hemolytic capacity, whereas 22 were oxidase negative and 1, L7-12, was oxidase-positive (Table 1). We then used the VITEK-MS system (bioMérieux, Durham, NC, USA) and sequencing of the *rrs* gene, which codes for the 16S rRNA subunit; all isolates (*n* = 23) were identified as *Vibrio metschnikovii*.

### 2.3. Antibiotic Susceptibility Profile

**Figure 1:**
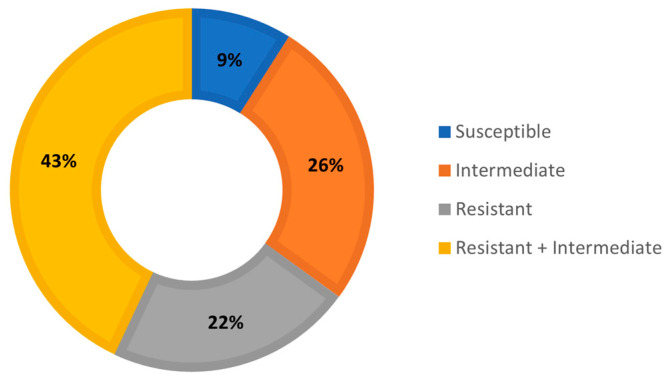
Percentages of sensitivity and resistance to antibiotics in *Vibrio* spp. isolates.

Notably, 9% of the isolates (*n* = 2) were susceptible to the antibiotics tested, whereas 22% (*n* = 5) showed resistance to cefotaxime (20%, *n* = 1), ceftazidime (20%, *n* = 1), and amikacin (60%, *n* = 3). Furthermore, 26% of the isolates (*n* = 6) presented reduced (intermediate) sensitivity to the different antibiotics tested, while a profile that combined resistance and reduced sensitivity to beta-lactams and aminoglycosides was observed in 43% of the isolates (*n* = 10) (Figure 1, Table 1).

No isolate showed resistance to multiple drugs; however, evaluation of the frequency of resistance for each antibiotic revealed that 56.5% (*n* = 13/23) were resistant to amikacin, 13% (*n* = 3/23) to ceftazidime, and 8.7% (*n* = 2/23) to each of the following antibiotics: cefotaxime and gentamicin (Table 1). All the isolates were sensitive to ciprofloxacin and imipenem (Table 1).

### 2.4. Genome Sequencing of Isolate L7-12

Isolate L7-12 was the only isolate that had a positive oxidase test result and showed simultaneous resistance to cefotaxime, ceftazidime, and amikacin. Thus, this isolate was selected for genome sequencing.

Average Nucleotide Identity (ANI) test results were used to determine that isolate L7-12 (accession number: JAUTDF010000000) showed 99% identity, with a coverage of >90%, to the type strains of *Vibrio injensis* (GCA_001895205.1). Conversely, it showed 91% identity, with 85% coverage, to the type strains of *Vibrio metschnikovii* (GCA_900460295.1).

Genes related to antibiotic resistance and virulence were detected through the genome sequence analysis of isolate L7-12. According to the results, this isolate carries the *bla*_CARB-9_ allele (99.2% identity), which codes for a beta-lactamase enzyme that—according to Ambler’s classification [24]—belongs to class A (Table 2). Another mechanism that contributes to antimicrobial resistance is efflux pumps, and—in this isolate—it was possible to identify the *nor*M gene, which codes for one that belongs to the MATE (Multi-Antimicrobial Extrusion) family. This expels drugs such as norfloxacin, among other toxic compounds (Table 2) and, finally, virulence factors associated with flagella synthesis (*fli*, *flh*), quorum sensing (*lux*), hemolytic activity (*tlh*, *tdh*, *hly*A), secretion proteins (*hcp*, *vgr*G-2), and intestinal colonization of the host (*gly*A-1) were detected. These give the bacteria pathogenic characteristics that facilitate their survival in different environments under conditions of stress (*rpo*S, *rpo*E), as well as help in establishing an infection in a host (Table 2).

## 3. Discussion

### 3.1. Identification of Isolates of Vibrio *spp.*

In this study, 23 isolates of sucrose-fermenting Gram-negative bacteria from two leachate lagoons were identified. These isolates were identified as *V. metschnikovii* using MALDI-TOF techniques and sequence analysis of the gene that codes for the 16S rRNA subunit. However, performing genome sequencing for isolate L7-12—which was selected as it was the only one to present a positive oxidase test and showed simultaneous resistance to cefotaxime, ceftazidime, and amikacin—revealed discrepancies in its identification when comparing the three methods used.

Bacteria were identified via MALDI-TOF-MS through the comparison of the characteristic spectrum of an unknown organism—known as the Peptide Mass Fingerprint (PMF)—with those available in the team’s database [25,26]. This tool was designed for clinical use; thus, its library mainly contains clinically relevant species [27], including different *Vibrio* species. Although the process is fast and sensitive, technology is limited with regard to the identification of new species or species that are not included in the library [26], as is the case of *V. injensis*. The BLASTn program looks for regions of local similarity between a 996 bp fragment corresponding to the *rrs* gene with other nucleotide sequences found in the base of NCBI data by calculating the statistical significance of matches (https://blast.ncbi.nlm.nih.gov/Blast.cgi, accessed on 22 June 2023). Furthermore, analysis of the partial sequence of the 16S rRNA gene using BLASTn allows the tool to infer functional and evolutionary relationships between them. Although the sequence similarity between the *rrs* gene of isolate L7-12 and that of *V. metschnikovii* is 100%, the fact that only the partial sequence of a gene is being compared should be taken into consideration. However, the ANI can discriminate between closely related species using WGS data, which is used to calculate the average similarity between homologous genomic regions shared between two genomes [28]. The results of the identification by ANI must consequently be considered more reliable; thus, we must identify isolate L7-12 as *V. injensis*.

Our results highlight the importance of contributing to websites, as it is not always possible to sequence the genomes of all the isolates that are part of a single research. These include PubMLST.org (https://pubmlst.org/about-us, accessed on 26 June 2023), which contains a collection of curated and open access databases with sequences for more than 100 bacterial species and genera [29], including an MLSA (Multi-Locus Sequence Analysis) scheme for *Vibrio* spp. [30]. However, no reports of loci or Type Sequences (ST) were found for *V. metschnikovii* isolates or *V. injensis* in PubMLST.org (https://pubmlst.org/organisms; accessed on 26 June 2023).

### 3.2. Antibiotic Susceptibility Profile of Vibrio *spp.* Isolates from Leachates

This is possibly the first study to report the presence of *V. metschnikovii* and *V. injensis* isolates in highly contaminating liquid matrices. Due to their composition, they are an ideal source for the growth of antibiotic-resistant bacteria [6,7]. Specifically, the results of this research corroborate that 22% of the *Vibrio* isolates were resistant to two of the families of antibiotics most used in clinical practice (third-generation cephalosporins and aminoglycosides; Table 1 and Figure 1); however, they are sensitive to imipenem (carbapenem) and ciprofloxacin (fluoroquinolone; Table 1). According to the CLSI, non-cholera species of the *Vibrio* genus are often resistant to sulfonamides, penicillins, and cephalosporins such as cephalothin and cefuroxime. Additionally, the results suggested that antibiotics—including third-generation cephalosporins (cefotaxime and ceftazidime), fluoroquinolones (ciprofloxacin), and tetracyclines—should be initially evaluated to treat infections that, except for the last one, were screened in this study.

Our results are consistent with those obtained by other authors who have recently isolated *V. metschnikovii* from aquatic environments [19,31,32,33,34]. Håkonsholm et al. found a Gram-negative bacterium on the west coast of Norway and reported that 21% of isolates were resistant to ampicillin (beta-lactam), whereas 13% were resistant to tobramycin (aminoglycoside) [31]. Valáriková et al. isolated *V. metschnikovii* from freshwater bodies in Slovakia and found that 100% of the isolates were resistant to penicillin (beta-lactam), while 90% were resistant to streptomycin (amino-glycoside) [19]. Furthermore, a study in South Africa on *V. metschnikovii* isolated from wastewater reported that 100% were resistant to ampicillin and cephalothin (beta-lactams), whereas 66% were resistant to amikacin (amino-glucoside) [32].

Likewise, the results corresponding to the antibiotic susceptibility profiles coincide with those of other researchers who have reported infections caused by *V. metschnikovii* both in animals and in humans. Flores et al. found this Gram-negative bacterium in fish captured in the Nicaraguan Pacific and reported that 100% of the isolates were resistant to amoxicillin/clavulanic acid, and 70% were resistant to ampicillin [33]. Moreover, *V. metschnikovii* isolates with reduced sensitivity to aminoglycosides, including amikacin and gentamicin, were identified in hybrid sturgeons cultivated in China [34]. However, Konechnyi et al. reported a case of infection caused by *V. metschnikovii* in a 70-year-old man in Ukraine. The antibiotic susceptibility profile included resistance to ceftazidime but susceptibility to aminoglycosides and quinolones [16]. No publications have reported antibiotic susceptibility profiles in *V. injensis* thus far.

Isolate L7-12 (Table 1) was selected to sequence its genome as previous reports by other authors indicated a high prevalence of resistance to beta-lactams and aminoglycosides in *Vibrio* [16,19,31,32,33,34]. When analyzing the results, the presence of the *bla*_CARB-9_ gene was detected (Table 2), which has been recently reported in *Vibrio metschnikovii* by Håkonsholm et al.; however, this has also been observed in other species such as *V. cholerae* [31,35,36], *V. alginolyticus*, and *V. antiquarius* [31]. The *bla*_CARB-9_ gene codes for a class A serino-beta-lactamase capable of hydrolyzing extended-spectrum carboxypenicillins [37] but not third-generation cephalosporins such as cefotaxime and ceftazidime. Neither integron, transposon, nor plasmid sequences were found to stablish the phenotype–genotype relationship for resistance to these antibiotics. Therefore, the hydrolytic profile of CARB-9 was not consistent with the results of the phenotypic susceptibility tests, which may be explained by the fact that the antibiotics could be expelled through efflux pumps, or because only 91.3% coverage of the sequenced genome was achieved. Similarly, although reduced susceptibility to aminoglycosides has been reported in *Vibrio* isolates [31,38,39], when sequencing the genome of isolate L7-12, no genes coding for acetyltransferases associated with this phenotypic profile were detected. Moreover, efflux pumps in the RND and MATE families that could be involved in the expulsion of aminoglycosides were identified [31,40,41,42].

These results suggested that complex matrices—such as leachates—are reservoirs of bacteria from different origins of solid waste, which may be resistant to different antimicrobial agents. Furthermore, leachates could provide the ideal conditions for the acquisition and dissemination of genes associated with antibiotic resistance due to the presence of antibacterial agents and biocides that exert selective pressure [19,43]. This, in addition to the horizontal transfer of intra- and inter-species resistance genes [19,44], as well as global warming, favors the increase in the population of *Vibrio* during warm-weather periods [19,45,46], as is the case in Cali for most of the year.

### 3.3. Virulence Factors in Isolate L7-12 of Vibrio injensis

Species in the *Vibrio* genus present virulence factors that increase their capacity to cause diseases in hosts [31,47], as well as to favor their survival in different environments.

Among the annotated genes that were part of the isolated virulome of *V. injensis* are those related to the production of hemolysins that affect the integrity of both erythrocytes and cell membranes (*tlh*, *tdh*, *hly*A, and *hly*D; Table 2), the incorporation of iron (*fur*), the biosynthesis of capsular polysaccharides that help resist opsonization and evade complement fixation (*wec*H), motility (*fla*, *fli*, *pil*) and flagella-mediated chemotaxis (*che*), the secretion system (T2SS), the production of secretion proteins (*hcp*, *vgr*G-2), and intestinal colonization of the host (*gly*A-1) (Table 2). Quorum-sensing genes (*eps*, *lux*) were also identified, which favor the formation of biofilms on biotic and abiotic surfaces [31,47,48,49,50,51,52] (Table 2).

Regarding the production of hemolysins, isolates of *V. alginolyticus*, *V. metschnikovii* and *V. anguillarum* carrying the *hly*A gene (*Vibrio cholerae* cytolysin A) have been reported [31], which is commonly found in *Vibrio cholerae* O1 and non-O1/non-O139 [19]. The *tlh* (thermolabile hemolysin) gene was observed in the species *V. fujianensis*, while—unlike in our study—none of the species found by Hakonshol et al. carried the *tdh* (thermostable direct hemolysin) gene [31]. HlyA has hemolytic and cytotoxic activity against different eukaryotic cells [31,53], whereas TDH is a toxin that forms pores in the erythrocyte membrane [54,55], which causes water and ions to flow across the membrane [54,56]. Hemolysins produced by *V. metschnikovii* are known to lyse cells from many animals including humans, sheep, and horses [31,57], and our results also suggest that all the isolates from the leachates have hemolytic capacity. Even though *V. injensis* can cause infections in humans, its virulome has been poorly studied; thus, the presence of these genes could suggest its pathogenic potential (Table 2) [58].

Iron is essential for the survival of organisms because it is involved in many processes ranging from cell signaling to metabolism [59,60]. Hence, bacteria have developed strategies that allow them to acquire iron from their hosts [59,61]. The process of iron uptake must be highly regulated because the iron concentration within the cell is critical, since too little of it prevents the development of some cellular processes, whereas too much iron causes the accumulation of free radicals, which would cause cell death [59,60]. In *V. parahaemolyticus*, this process is controlled by the iron uptake regulator protein Fur (Ferric Uptake Regulator; Table 2) [59,62]. Furthermore, low intracellular iron concentrations stimulate the expression of virulence genes in bacteria such as *V. cholerae* [59,63]. The expression of the *hly*A gene was reported to be regulated by Fur (among other regulators, such as HapR and HlyU; Table 2), which favors the invasion of the host by this bacterium, even though the pathogenesis of invasive infections caused by *V. cholerae* is not fully known [64].

However, for *V. vulnificus*, the synthesis of capsular polysaccharides (*wec*H, Table 2) plays a fundamental role in the evasion of the host’s innate immune system by conferring antiphagocytic capacity and resistance to destruction mediated by the complement system [65].

Motility plays an important role in species of the *Vibrio* genus, either in the aquatic environments in which they live or during host colonization. Regarding *V. cholerae*, motility occurs through polar flagella, while in *V. parahaemolyticus*, it occurs through polar and lateral flagella, whose regulation is very complex and depends on the hierarchical expression of genes grouped into the following four classes: I (*flr*A), II (*flh*A, *fli*EGI, *fli*R, *fli*A), III (*flh*B, *fli*MNQ), and IV (*fla*BD) (Table 2) [66]. The colonization of hosts by *V. cholerae* was reported to require a functional flagellum to bind to the intestinal epithelium and colonize it, after which the flagellum is lost [64]. The loss of the flagellum triggers a series of regulatory events, among which is the production of sigma factor, FliA, which promotes the expression of the gene that encodes the HlyA toxin (among others, such as CT and Tcp) [64,67], as well as the type 6 secretion apparatus (T6SS, Table 2) [64,68]. This leads to the increased hemolysis of human erythrocytes [64]. Furthermore, an operon with five genes associated with bacterial chemotaxis (*che*Y3, *che*Z, *che*AB, and *che*W1; Table 2) is known to exist, and hypervirulent strains of *V. cholerae* have been shown to have high levels of *flh*A expression but low expression of *che*W1 (Table 2). This means that the flagellar and chemotaxis gene pools are regulated differently. Therefore, during the disease process, flagellar genes briefly repress chemotaxis genes and maintain motility to accelerate infectivity [64].

Additionally, computational analysis revealed that 30% of intestinal bacteria can produce type IV pilus (T4P), which is characterized as an extracellular filament composed of PilA subunits (Table 2), which are helically assembled. In Gram-negative bacteria, the outer membrane pore through which the filament passes is made up of the PilQ protein (Table 2), whereas PilT (Table 2) is an ATPase that is involved in pili retraction. T4P was reportedly related to adhesion, biofilm formation, twitching motility, and the horizontal transfer of genetic information [69]. However, in *V. cholerae*, mannose-sensitive hemagglutinin (MSHA, Table 2) is also a member of the T4P family and has been described as a host colonization factor and a mediator in the transfer of DNA in Gram-negative bacteria [64]. T4Ps are evolutionarily related to T2SS, as they share a similar structure and functionality [69,70]. In *V. cholerae*, T2SS, also known as the extracellular protein secretion (Eps) system (*gsp*k, *gsp*F; Table 2) is involved in the transport of hydrolytic enzymes which are translocated through the outer membrane [64,71]. Specifically, in the *V. injensis* isolate L7-12, the *hcp* and *vgr*G-2 genes were annotated, which code for secretion proteins related to survival and the destruction of competing or predatory microorganisms through perforation [72].

Finally, quorum sensing (QS) needs to occur for the formation of biofilms, which is involved in not only their composition but also the synchronization of other bacterial functions such as the expression of virulence genes, production of secondary metabolites, and destruction of competition through secretion systems. In *V. cholerae*, a threshold concentration of the autoinducers is not reached when there is low cell density, so the kinases LuxPQ and CqsS sequentially phosphorylate LuxO and LuxU (Table 2). Once the RNA polymerase binds to the sigma factor 54, LuxO starts transcribing the genes that code for the ncRNA Qrr regulators which, in turn, activate the translation of AphA; in contrast, they repress HapR translation with Hfq. These conditions promote the expression of genes encoding virulence factors and the formation of biofilms [64].

In summary, the genes associated with virulence factors that were annotated for the *V. injensis* isolate are related to its pathogenic potential through antimicrobial resistance, as well as to its virulence. Different studies in other *Vibrio* species have shown that, of all the virulence factors, flagellar motility seems to play an important role in pathogenesis because it participates in the formation of biofilms [66,73] that allow it to adapt and survive in host cells, as well as to different aquatic environmental conditions [66,74,75]. Furthermore, although the flagellum is crucial for motility, the main appendages used for the initial attachment of this bacterium to the host are T4Ps, such as mannose-sensitive hemagglutinin (MSHA) [66,76]. Moreover, flagellar motility is associated with various cellular processes, such as colonization [66].

## 4. Materials and Methods

### 4.1. Sampling and Collection Sites of Leachate Samples

**Figure 2:**
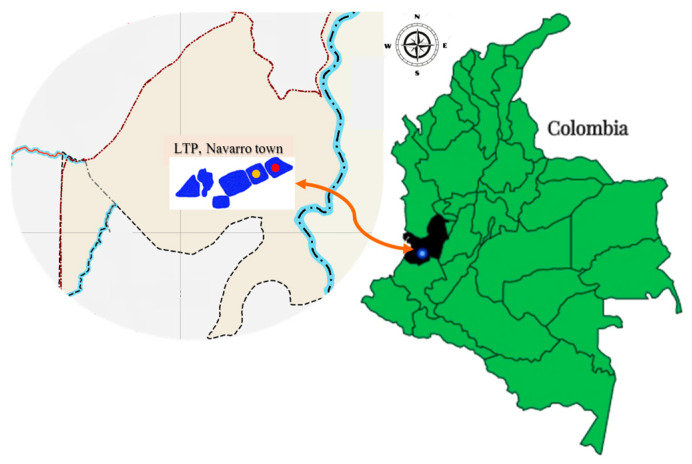
Location of the leachate treatment plant (LTP) in Navarro town, Santiago de Cali municipality (blue dot), Valle del Cauca department (black), and the sampling location: Lagoon 3 (yellow dot); Lagoon 7 (red dot).

This is a descriptive study with simple and intentional sampling, wherein selective and differential culture media were used that allowed for the selection of *Vibrio* spp. isolates from several leachate samples collected from a leachate treatment plant (LTP) located in the town of Navarro (3°23′13.8″ N 76°29′7.5″ W, Figure 2), Municipality of Santiago de Cali, Colombia (Figure 2). The treatment plant comprises a pumping station (PS), which distributes the leachate from the landfill to seven lagoons, five of which were treated using physicochemical methods while no treatment was applied to the remaining two (Lagoons 3 and 7). Samples were collected in March 2019.

The sample collection methods and treatments of the collected samples have been described in our previous work [77]. A 200 mL sample was collected from each of Lagoons 3 and 7, which was diluted at a 1:20 ratio in SS (1%). Subsequently, 100 mL was filtered through a cellulose–ester membrane (0.45 µm; Merck Millipore, Germany) using vacuum filtration equipment (Merck Millipore, Darmstadt, Germany). The filter was transferred to Thiosulfate Citrate Bile Sucrose Agar (TCBS, Oxoid, Basingstoke, UK) plates, which were incubated for 18–20 h at 37 °C. Yellow, sucrose-fermenting colonies grown on this medium were considered presumptive *Vibrio* spp. and were reisolated on TSA plates (Oxoid, Basingstoke, UK) for later identification.

### 4.2. Biochemical Tests and Identification of Isolates of Vibrio *spp.*

The 23 presumed *Vibrio* spp. isolates that grew in TSA underwent Gram staining [78] and the oxidase test (reference 55635, *bioMérieux*, Durham, NC, USA). In addition, their hemolytic capacity was determined, for which they were seeded on Columbia Blood Agar plates supplemented with 5% sheep blood (reference 43049, *bioMérieux*, Durham, NC, USA) and incubated at 37 °C for 24 h. The *E. coli* strain ATCC 25922 was used as the negative control in the test.

The 23 presumed *Vibrio* spp. isolates were identified using MALDI-TOF MS (Matrix-Assisted Laser Desorption/Ionization–Time-Of-Flight; VITEK^®^MS *bioMérieux*, Durham, NC, USA), following the manufacturer’s instructions. To confirm the results, they were identified through sequence analysis of the gene that codes for the 16S ribosomal RNA subunit (16S rRNA). DNA extraction was performed by resuspending a colony of the isolate in 100 μL of sterile distilled water. The bacteria were lysed using boiling water for 10 min. Cell debris was removed via centrifugation at 13,000 rpm for 5 min, and the supernatant was used as the template DNA for the polymerase chain reaction. For this, the U1 and U2 primers [79] (996 bp) and the PCR 100 2× mix (CorpoGen, OPTBM-00006, Bogotá, Colombia) were used. After amplification, the fragments were purified using the commercial Qiaquick PCR Spin kit (Qiagen, Hilden, Germany) and sequenced with the same primers used for PCR amplification (Macrogen, Seoul, Korea). To examine the obtained sequences, the ChromasPro program (version 2.1.8) was used, while the BLAST tools were employed to carry out the analysis of the sequences and to identify the isolates. (Basic Local Alignment Search Tool; https://blast.ncbi.nlm.nih.gov/Blast.cgi, accessed on 24 April 2022) and RDP (The Ribosomal Database Project; https://rdp.cme.msu.edu/, accessed on 24 April 2022).

### 4.3. Antibiotic Susceptibility Profile of the Isolates of Vibrio *spp.*

The antibiotic susceptibility tests for the identified isolates were performed using the Kirby–Bauer disk diffusion method, and the interpretation was according to the 2016 M45 guide of the Clinical and Laboratory Standards Institute (CLSI) [80]. For this, an isolated bacterial colony was collected and diluted in sterile saline solution (0.85%) until it reached a turbidity of 0.5 on the McFarland scale. The bacterial suspension was seeded in bulk on Müller Hinton (MH) agar plates (reference 413822, *bioMérieux*, Durham, NC, USA) using a sterile swab. Subsequently, the antibiotic disks were placed onto the plates, and the plates were incubated at 37 °C for 18–20 h. Each bacterial isolate was tested against six antibiotics commonly used in clinical practice that belong to the following three families: (i) beta-lactams: ceftazidime (30 µg), cefotaxime (30 µg), and imipenem (10 µg); (ii) fluoroquinolones: ciprofloxacin (5 µg); and (iii) aminoglycosides: amikacin (30 µg) and gentamicin (10 µg) (Oxoid™). The inhibition halos were measured with a Vernier scale and compared with the reference frames according to the M45 guide provided by the CLSI [80]. *E. coli* ATCC 25922 was used as a sensitivity control, and the M100-2023 guide was used to interpret the results of the reference strain [81].

### 4.4. Genome Sequencing of Isolate L7-12

The L7-12 isolate was selected for genome sequencing and gDNA extraction was performed using the MagNA Pure 96 automated extraction system (Roche, Penzberg, Germany) following the manufacturer’s instructions. To verify the integrity of the gDNA, a horizontal electrophoretic run was performed on an agarose gel (0.8%) and the DNA was quantified using the NanoDrop ND-1000 (NanoDrop Technologies, Wilmington, DE, USA). DNA was sequenced by Novogene Corporation Inc. (Durham, NC, USA) using the NovaSeq6000 platform (Illumina Inc., San Diego, CA, USA). The quality of the raw sequence was evaluated using the FastQC tool (version 0.11.9) (Babraham Bioinformatics—FastQC: a Quality Control tool for High Throughput Sequence Data, n.d.), and the adapters were subsequently removed with FastClipper (fastx_toolkit, version 0.0.14). Additionally, sequence filtering was performed using Fastp [82]. The genome was assembled with SPAdes [83] and evaluated with QUAST [84]. For gene annotation, the CGE-ResFinder platforms were used [85], i.e., BV-BRC [86] and Kbase-Prokka [87]. This whole genome shotgun project has been deposited at DDBJ/ENA/GenBank under the accession number JAUTDF000000000. The version described in this paper is version JAUTDF010000000.

## 5. Conclusions

To the best of our knowledge, the antibiotic susceptibility profile of *Vibrio* species isolated from leachates in Colombia has not been reported before. The bacterial susceptibility to antimicrobial agents also fluctuates spatially and temporally because natural environments are dynamic. Thus, the importance of the continuous monitoring and surveillance of these susceptibilities in *Vibrio* species is highlighted to better understand the local epidemiology, not only focusing on human health but also on animal and environmental health [32]. Our results demonstrate that leachates could be reservoirs of the *Vibrio* species with pathogenic potential, not only due to the production of virulence factors, but also thanks to the presence of antibiotic-resistance determinants, which highlights the importance of the proper operation of a leachate treatment plant. Furthermore, it is necessary to specify the identification of the *Vibrio* species to strengthen decision making related to infection control measures both in humans and in other animals. This is to learn about the resistance and virulence mechanisms associated with the species *V. injensis*, since most of the information that is available corresponds to *V. cholerae* and *V. parahaemolyticus*. Finally, this is the first report of the draft-genome of *V. injensis* that indicates the pathogenic potential of this species in Colombia.

## Figures and Tables

**Table 1:** Oxidase test and antibiotic susceptibility profile in *Vibrio* spp. isolates.

Sampling Site	Isolate Name	Oxidase Test	Antibiotic Susceptibility Profile (Kirby–Bauer Test)					
			CTX	CAZ	IPM	CIP	AM	CN
**Lagoon 3**	**L3-2**	−	S	S	S	S	R	I
	**L3-5**	−	S	S	S	S	R	S
	**L3-7**	−	S	S	S	S	I	I
	**L3-18**	−	R	S	S	S	S	S
	**L3-19**	−	S	S	S	S	S	S
**Lagoon 7**	**L7-1**	−	S	S	S	S	R	I
	**L7-2**	−	S	S	S	S	R	S
	**L7-3**	−	S	S	S	S	I	S
	**L7-4**	−	S	R	S	S	R	I
	**L7-5**	−	S	S	S	S	R	I
	**L7-6**	−	S	S	S	S	I	S
	**L7-7**	−	S	S	S	S	R	I
	**L7-9**	−	S	S	S	S	R	S
	**L7-10**	−	S	I	S	S	R	R
	**L7-11**	−	S	S	S	S	I	S
	**L7-12**	+	R	R	S	S	R	I
	**L7-13**	−	S	I	S	S	R	S
	**L7-14**	−	S	S	S	S	R	I
	**L7-15**	−	S	R	S	S	S	S
	**L7-16**	−	S	S	S	S	S	S
	**L7-17**	−	I	I	S	S	R	R
	**L7-18**	−	S	I	S	S	I	S
	**L7-20**	−	I	S	S	S	S	S

Abbreviations: CTX: Cefotaxime; CAZ: Ceftazidime; IMP: imipenem; CIP: Ciprofloxacin; AM: Amikacin; CN: Gentamicin; S: Sensitive; R (dark gray): Resistant; I (light grey): Intermediate; +: Positive (oxidase positive); −: Negative (oxidase negative). Yellow indicates the isolates from lagoon 3, while red indicates those from lagoon 7.

**Table 2:** Gene annotation of *Vibrio injensis* isolate L7-12.

Genes	Functions
Sigma factors	
*rpo*S	Survival due to nutritional, oxidative, and osmotic stress
*rpo*E	
Antibiotic resistance	
*bla*_CARB-9_	Carbenicillin (Beta-lactam) resistance
Efflux pumps	
*nor*M	MATE
*ade*F, *ade*G, *ade*H	RND
Virulence factors	
*tlh*, *tdh*, *hly*A, *rxtC*	Hemolysins
*fur*, *vib*B	Iron uptake
*wec*H	Capsular antiphagocytosis polysaccharides
*gly*A-1	Intestinal colonization
*flh*A, *fli*EGI, *fli*R, *fli*A, *flh*B, *fli*MNQ, *fla*BD, *fle*N	Flagellar synthesis
*che*Y2, *che*Z, *che*AB, *che*W1	Chemotaxis
*pil*A, *pil*Q, *pil*T	Type IV pilus
*msh*A	Mannose-sensitive Hemagglutinin
*gsp*k, *gsp*F, *hcp*, *vgr*G-2	Extracellular protein secretion
*lux*PQ, *lux*O, *lux*U	Quorum sensing

## Data Availability

The data used to support the findings of this study are included within the article and are available from the corresponding author upon request. This Whole Genome Shotgun project has been deposited at DDBJ/ENA/GenBank under the accession JAUTDF000000000. The version described in this paper is version JAUTDF010000000.
